# Changes in Circulating Acylated Ghrelin and Neutrophil Elastase in Diabetic Retinopathy

**DOI:** 10.3390/medicina60010118

**Published:** 2024-01-08

**Authors:** Maria Consiglia Trotta, Carlo Gesualdo, Marina Russo, Caterina Claudia Lepre, Francesco Petrillo, Maria Giovanna Vastarella, Maddalena Nicoletti, Francesca Simonelli, Anca Hermenean, Michele D’Amico, Settimio Rossi

**Affiliations:** 1 Department of Experimental Medicine, University of Campania “Luigi Vanvitelli”, 80138 Naples, Italy; mariaconsiglia.trotta2@unicampania.it (M.C.T.); caterinaclaudia.lepre@unicampania.it (C.C.L.); francesco.petrillo@unicampania.it (F.P.); michele.damico@unicampania.it (M.D.); 2 Multidisciplinary Department of Medical, Surgical and Dental Sciences, University of Campania “Luigi Vanvitelli”, 80138 Naples, Italy; carlo.gesualdo@unicampania.it (C.G.); mnicoletti2023@libero.it (M.N.); francesca.simonelli@unicampania.it (F.S.); 3 PhD Course in National Interest in Public Administration and Innovation for Disability and Social Inclusion, Department of Mental, Physical Health and Preventive Medicine, University of Campania “Luigi Vanvitelli”, 80138 Naples, Italy; marina.russo@unicampania.it; 4 School of Pharmacology and Clinical Toxicology, University of Campania “Luigi Vanvitelli”, 80138 Naples, Italy; 5 PhD Course in Translational Medicine, University of Campania “Luigi Vanvitelli”, 80138 Naples, Italy; mariagiovanna.vastarella@unicampania.it; 6 “Aurel Ardelean” Institute of Life Sciences, Vasile Goldis Western University of Arad, 310144 Arad, Romania; hermenean.anca@uvvg.ro

**Keywords:** diabetic retinopathy, ghrelin, neutrophils, neutrophil extracellular traps

## Abstract

*Background and Objectives*: The role and the levels of ghrelin in diabetes-induced retinal damage have not yet been explored. The present study aimed to measure the serum levels of total ghrelin (TG), and its acylated (AG) and des-acylated (DAG) forms in patients with the two stages of diabetic retinopathy (DR), non-proliferative (NPDR) and proliferative (PDR). Moreover, the correlation between serum ghrelin and neutrophil elastase (NE) levels was investigated. *Materials and Methods*: The serum markers were determined via enzyme-linked immunosorbent assays in 12 non-diabetic subjects (CTRL), 15 diabetic patients without DR (Diabetic), 15 patients with NPDR, and 15 patients with PDR. *Results*: TG and AG serum levels were significantly decreased in Diabetic (respectively, *p* < 0.05 and *p* < 0.01 vs. CTRL), NPDR (*p* < 0.01 vs. Diabetic), and in PDR patients (*p* < 0.01 vs. NPDR). AG serum levels were inversely associated with DR abnormalities (microhemorrhages, microaneurysms, and exudates) progression (r = −0.83, *p* < 0.01), serum neutrophil percentage (r = −0.74, *p* < 0.01), and serum NE levels (r = −0.73, *p* < 0.01). The latter were significantly increased in the Diabetic (*p* < 0.05 vs. CTRL), NPDR (*p* < 0.01 vs. Diabetic), and PDR (*p* < 0.01 vs. PDR) groups. *Conclusions*: The two DR stages were characterized by decreased AG and increased NE levels. In particular, serum AG levels were lower in PDR compared to NPDR patients, and serum NE levels were higher in the PDR vs. the NPDR group. Together with the greater presence of retinal abnormalities, this could underline a distinctive role of AG in PDR compared to NPDR.

## 1. Introduction

Diabetic retinopathy (DR) represents the main cause of blindness, especially in industrialized countries, with a significant impact on healthcare costs [1]. As one of the more frequent diabetes complications, DR is present in up to 50% of patients with type I diabetes after 30 years from diabetes diagnosis, and in 12–19% of patients with type II diabetes at the time of diabetes diagnosis [2,3,4]. Although intravitreal injections of anti-VEGF drugs and steroids seem to be effective for the management of both non-proliferative (NPDR) and proliferative (PDR) DR forms [5,6], DR detection is still a challenge since it requires complex and expensive equipment, along with well-trained technicians [7]. Therefore, the goal of recent DR studies is to develop innovative approaches aimed at providing an early identification of the disease and improving DR care. A very interesting and promising tool could be represented by the use of artificial intelligence (AI) in DR screening for diabetic children, which seems to be effective and cost-saving [4]. In addition, the identification of new systemic biomarkers correlated to the stage of the disease, and involved in its evolution, could be a useful novel tool for the prediction or early identification of NPDR in elderly people and to slow its progression to PDR [8].

In this regard, a retinal protective role has been attributed to ghrelin, a peptide hormone produced by the pancreatic islet in the fetal period and mainly deriving from gastrointestinal endocrinal cells (P/D1 cells in the oxyntic mucosa of the stomach and upper small intestine) in adult life, as well as from pancreatic epsilon cells and the hypothalamus [9,10,11,12,13]. Beyond its role as an orexigenic signal [14], ghrelin has been reported to mediate anti-apoptotic, autophagic, and anti-inflammatory effects at the level of the eye structures, for example, in the epithelial cells of the lens membrane [15] and in the retina [16]. Interestingly, previous preclinical studies also evidenced a protective role of ghrelin in DR in vitro and in vivo models [17,18]. Specifically, ghrelin was shown to prevent cell death and to reduce pro-inflammatory interleukins in human retinal epithelial cells (ARPE-19) and in human retinal microvascular endothelial cells (HMRECs) exposed to high glucose [17,18]. Moreover, ghrelin was found to reduce blood glucose levels and to prevent morphological alterations in retinal layers in diabetic rats, by attenuating retinal cell apoptosis and the formation of degenerate capillaries [17]. 

Worthy of note is that HMRECs express the ghrelin receptor, known as growth hormone secretin receptor 1a (GHSR-1a), characterized by its higher affinity to the acylated form of ghrelin compared to the des-acylated one [18,19]. GHSR-1a is also expressed by immune cells, including neutrophils [20,21,22,23,24]. These circulating cells play a very important defensive role in the first steps of hyperglycemia-induced acute inflammation, via the degranulation, phagocytosis, and extrusion of neutrophil extracellular traps (NETs) [25]. However, during chronic inflammation, the exacerbated presence of neutrophils and NETs is associated with the thickening of the retinal vascular basement membrane, loss of pericytes, and alterations in the blood–retinal barrier, which, combined, trigger DR microvascular and macrovascular complications [25,26,27,28,29,30,31]. Interestingly, by binding to GHSR-1a on neutrophils, ghrelin is able to promote their apoptosis [22,23], potentially impacting DR microvascular complications in diabetic patients.

Therefore, the present study aimed to evaluate in humans whether there are putative changes in total levels of serum ghrelin and/or its acylated/des-acylated forms during different phases of DR, and whether they correlate with clinical signs and serum neutrophil elastase levels, indicative of NET formation [20] and vascular damage [29,32,33].

## 2. Materials and Methods

### 2.1. Clinical Design

This study was performed at the Eye Clinic of the University of Campania “Luigi Vanvitelli” (Naples, Italy), with all its procedures adhering to the Declaration of Helsinki and Good Clinical Practice guidelines. The study was approved by the ethics committee of the University of Campania “Luigi Vanvitelli” (protocol number 42 DEC, 30 January 2019 and protocol number 0003239/i, 1 February 2023). The patients were enrolled after a basal examination, which was needed to evaluate the adherence to the following inclusion criteria: (I) age of at least 46 years; (II) duration of diabetes of at least 4 years; (III) clinical diagnosis of NPDR or PDR confirmed using medical history and ocular fundus examination; and (IV) hypoglycemic therapy with sodium–glucose cotransporter 2 inhibitors [34]. Moreover, written informed consent was obtained from all the participants, who read and signed the document after receiving clear explanations about the nature of the study and its possible consequences. Patients with a body mass index higher than 30 kg/m^2^ (obese patients), uncontrolled diabetes, other severe diabetic complications (ketoacidosis/nephropathy), recent or systemic infection, or recent cardiovascular diseases were excluded. Furthermore, patients using lipid-lowering agents or immunosuppressive, steroidal, and non-steroidal anti-inflammatory drugs (NSAIDs) were not enrolled, nor were patients who had received intravitreal steroids/anti-vascular endothelial growth factors (VEGFs), or argon laser coagulation or vitrectomy in the last 6 months.

### 2.2. DR Diagnosis

DR diagnosis was performed by obtaining the patient’s medical history and by performing an ocular fundus examination. DR stages were determined by the number of intraretinal microvascular abnormalities, microaneurysms, hemorrhages, and retinal neovascularization, according to the American Academy of Ophthalmology Retina-Vitreous Panel [35]. This clinical classification provides the distinction between two large DR stages, NPDR and PDR. NPDR is characterized by at least one microaneurysm (mild NPDR), microhaemorrhages, exudates, and venous beading (moderate NPDR form), and finally up to >20 microhaemorrhages spread in the four quadrants and IRMA (severe NPDR form). On the other hand, the PDR form is characterized by the presence of retinal new vessels, or pre-retinal or vitreous haemorrhages with fibrovascular proliferation. Retinography analysis was performed by using Topcon Engineering.

### 2.3. Serum Sample Collection and Analysis of Serum Markers

Sterile dry vacutainer tubes were used to obtain fasting venous blood samples from the enrolled patients. Within 2 h after sampling, blood was incubated at 20 °C for 30 min before being centrifuged at 4 °C for 15 min at 3000 rpm, to have serum samples as supernatants. Their aliquots were stored at −80 °C for subsequent analysis of serum markers, performed at the Pharmacology Section of University of Campania “Luigi Vanvitelli”) through enzyme-linked immunosorbent assays (ELISAs). Serum acylated ghrelin, total ghrelin, and neutrophil elastase levels were assessed by using commercial ELISAs according to the manufacturers’ instructions (respectively, EH2601 Human Acylated Ghrelin ELISA Kit, FineTest—Wuhan, China; EH0355 Human Ghrelin ELISAS Kit, FineTest—Wuhan, China; and BMS269 Human PMN-Elastase ELISA Kit, Invitrogen—Waltham, MA, USA). These kits were based on sandwich enzyme-linked immune-sorbent assay technology, with primary antibodies pre-coated on the 96-well plates and detected by using specific biotin-labeled secondary antibodies. Briefly, standards and samples were subsequently added to the plates and incubated according to the different manufacturers’ protocols. Unbound conjugates were removed by using wash buffer, then biotinylated secondary antibodies were added to the wells. Again, unbound conjugated were washed off before adding horseradish peroxidase (HRP)-streptavidin conjugate, by avoiding direct light. After a third washing, HRP enzymatic reactions were visualized by adding the 3,3′,5,5′-Tetramethylbenzidine (TMB) substrate, catalyzed by HRP to produce a blue color product. This turned into yellow after the reaction was stopped. At this point, optical density (OD) absorbance was read using a microplate reader at the wavelength indicated by the manufacturers’ protocols, to obtain a standard curve and calculate the concentrations of the serum markers. Serum des-acylated ghrelin levels were calculated by subtracting acylated form from total ghrelin [36]. Neutrophil percentage was obtained from the leukocyte formula.

### 2.4. Statistical Analysis

Prism 6.0 (GraphPad, San Diego, CA, USA) software was used for both statistical analysis and graph design. One-way analysis of variance (ANOVA), followed by a Tukey multiple comparison post hoc test, were used for the determination of differences between groups. The strength of association between two quantitative variables was evaluated via Pearson correlation analysis; DR stages were categorized with a DR severity scale, as previously reported [37,38]. This was modified by grouping all NPDR forms into one NPDR stage, as follows: 1 = no retinopathies, no diabetes; 2 = no retinopathies, diabetes; 3 = NPDR forms; 4 = PDR. For both ANOVA and Pearson correlation, a *p*-value < 0.05 was considered significant. 

## 3. Results

### 3.1. Characteristics of DR Patients

A total of 57 patients (31 males and 26 females) were enrolled in this study and divided into the following four groups, based on ocular evaluations and DR clinical diagnosis:

I. Non-diabetic subjects with absence of ocular pathologies (*n* = 12)—CTRL group;
II. Diabetic patients without DR signs (*n* = 15)—Diabetic group;
III. Diabetic patients with diagnosis of non-proliferative DR (*n* = 15)—NPDR group;
IV. Diabetic patients with diagnosis of proliferative DR (*n* = 15)—PDR group.

Differences in age or diabetes duration were not observed in the diabetic groups. Conversely, PDR patients exhibited significantly higher glycaemic levels compared to the Diabetic and NPDR groups (both *p* < 0.05) (Table 1). 

Moreover, the neutrophil percentage significantly increased in NPDR patients compared to the Diabetic group (*p* < 0.01). This was even more elevated in PDR patients (*p* < 0.01 vs. NPDR) (Table 1), although the values for all four clinical groups were in the normal range. 

### 3.2. Serum Ghrelin Levels in DR Patients

**Figure 1:**
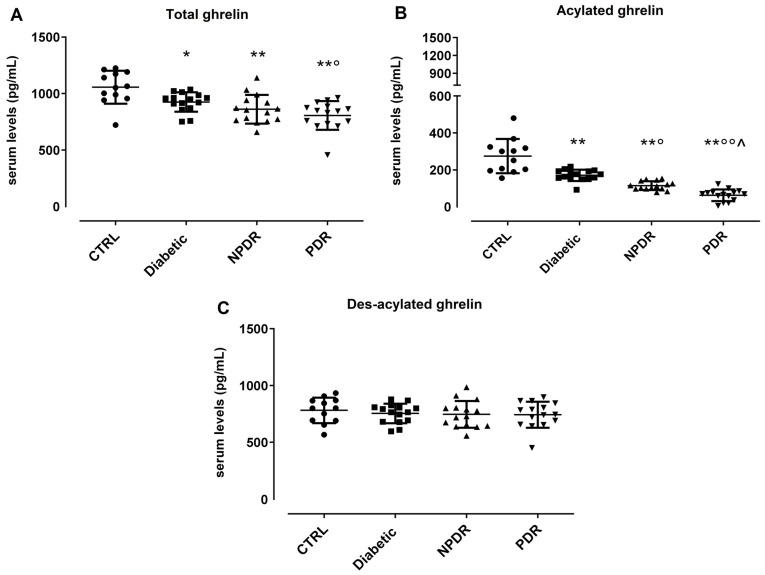
Serum levels of (**A**) total ghrelin (pg/mL ± SD), (**B**) acylated ghrelin (pg/mL ± SD), and (**C**) des-acylated ghrelin (pg/mL ± SD) in non-diabetic subjects with absence of ocular pathologies (*n* = 12, CTRL); diabetic patients with no signs of diabetic retinopathy (*n* = 15, Diabetic); diabetic patients with non-proliferative diabetic retinopathy (*n* = 15, NPDR) or proliferative retinopathy (*n* = 15, PDR); * *p* < 0.05 and ** *p* < 0.01 vs. CTRL; ° *p* < 0.05 and °° *p* < 0.01 vs. Diabetic; ^ *p* < 0.05 vs. NPDR.

The serum total ghrelin (TG) was significantly decreased in the Diabetic group compared to CTRL subjects (CTRL: 1056 + 146 pg/mL; Diabetic: 925 + 86 pg/mL, *p* < 0.05 vs. CTRL). A reduction in serum TG levels, although not significant in comparison with the Diabetic group, was detected also in the NPDR group (NPDR: 861 + 126 pg/mL, *p* > 0.05 vs. Diabetic), while it was significant in PDR patients compared to the Diabetic group (PDR: 807 + 127 pg/mL, *p* < 0.05 vs. Diabetic) (Figure 1A).

Similarly, serum acylated ghrelin (AG) was significantly decreased in the Diabetic group compared to CTRL subjects (CTRL: 275 ± 93 pg/mL; Diabetic: 171 ± 31 pg/mL, *p* < 0.01 vs. CTRL). A further significant reduction was evident in NPDR patients compared to the Diabetic group (NPDR: 115 ± 24 pg/mL, *p* < 0.05 vs. Diabetic), with the lowest serum AG levels detected in PDR sera (PDR: 63 ± 32 pg/mL, *p* < 0.01 vs. NPDR) (Figure 1B). Conversely, serum des-acylated ghrelin (DAG) was not differentially modulated in our clinical setting (CTRL: 800 ± 182 pg/mL; Diabetic: 926 ± 165 pg/mL; NPDR: 917 ± 248 pg/mL; PDR: 990 ± 193 pg/mL) (Figure 1C). The serum TG, AG, and DAG levels in the male and female subgroups are reported in Figure S1.

### 3.3. Serum AG/DAG Ratio and Its Correlation with Retinal Abnormalities in DR Patients

**Figure 2:**
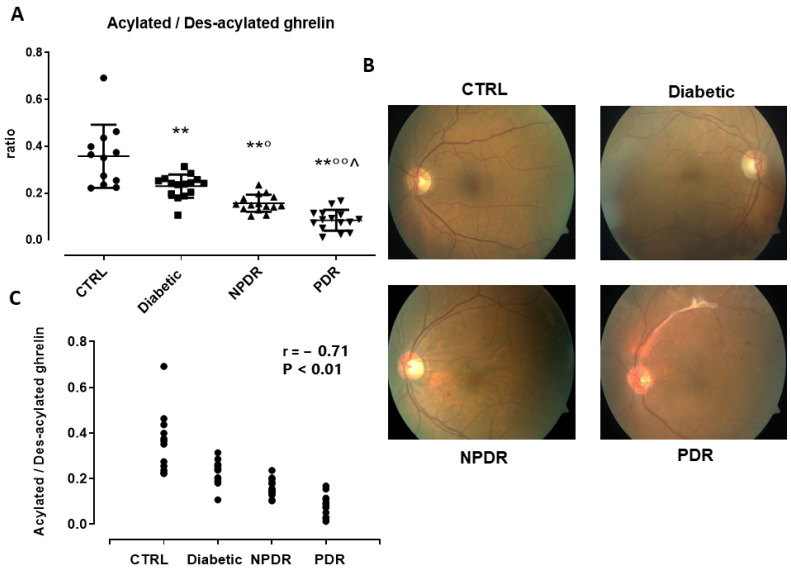
(**A**) Serum acylated/des-acylated ratio ± SD in non-diabetic subjects with absence of ocular pathologies (*n* = 12, CTRL); diabetic patients with no signs of diabetic retinopathy (*n* = 15, Diabetic); diabetic patients with non-proliferative diabetic retinopathy (*n* = 15, NPDR); and those with proliferative retinopathy (*n* = 15, PDR); (**B**) Representative retinography images of CTRL (normal retinal vasculature), Diabetic (slight irregularity in the caliber and vessel course, without DR signs) NPDR (multiple microhemorrhages, microaneurysms, and exudates in the macular region), and PDR groups (multiple microhemorrhages with extensive fibrovascular proliferation along the superior temporal vascular arch); (**C**) Pearson correlation coefficient (r = −0.83) and significance level (*p* < 0.01) for the correlation of the acylates/des-acylated ratio and DR stages in the four study groups. ** *p* < 0.01 vs. CTRL; ° *p* < 0.05 and °° *p* < 0.01 vs. Diabetic; ^ *p* < 0.05 vs. NPDR.

Consequently, the AG/DAG ratio was significantly decreased in Diabetic patients (0.23 ± 0.05, *p* < 0.01 vs. CTRL) compared to the CTRL group (0.35 ± 0.1). A significant reduction of AG/DAG ratio was evident also in NPDR (0.16 ± 0.04, *p* < 0.05 vs. Diabetic CTRL) and PDR (0.08 ± 0.04, *p* < 0.01 vs. Diabetic and *p* < 0.05 vs. NPDR) patients (Figure 2A). 

The decrease in AG/DAG was paralleled by an increase in the microhemorrhages, microaneurysms, and exudates detected in NPDR and PDR patients, respectively, via retinography. Indeed, CTRL subjects evidenced a normal retinal vasculature without abnormalities, and Diabetic patients showed a slight irregularity in the caliber and vessel course without DR classic signs (Figure 2B). Conversely, NPDR patients evidenced multiple microhemorrhages (1− ≥20), microaneurysms, and exudates in the four retinal quadrants, while the PDR group showed retinal neovascularization, and/or extensive fibrovascular proliferation, or vitreous/preretinal hemorrhages. This increase in retinal abnormalities identifying the DR stage was significantly inverse-correlated with the serum AG/DAG ratio (r = −0.83, *p* < 0.01) (Figure 2C). The AG/DAG ratios in the male and female subgroups are reported in Figure S2, along with their correlation with the DR stage.

Therefore, since the modulation of the AG/DAG ratio in DR patients was due to serum AG changes, this was considered for further correlations. 

### 3.4. Serum Neutrophil Elastase (NE) Levels in DR Patients and Their Association with Serum AG

**Figure 3:**
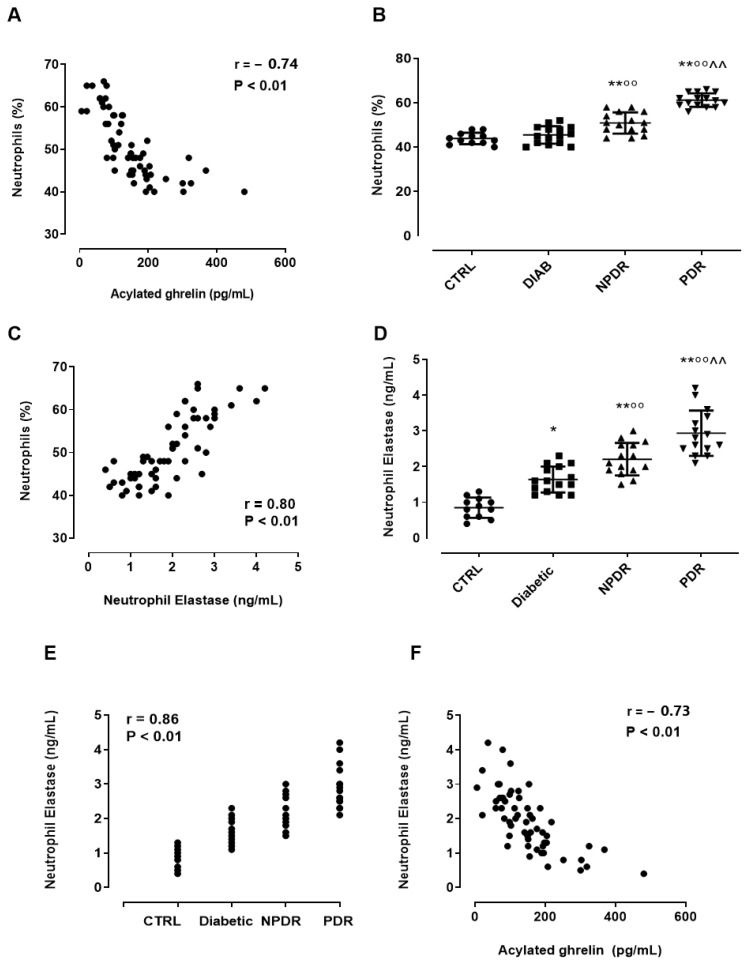
(**A**) Pearson correlation coefficient (r) and significance level (*p*) for the correlation of the neutrophil percentage (%) with serum AG levels (PG/mL ± SD; r = −0.74, *p* < 0.01) in non-diabetic subjects with absence of ocular pathologies (*n* = 12, CTRL); diabetic patients with no signs of diabetic retinopathy (*n* = 15, Diabetic); diabetic patients with non-proliferative diabetic retinopathy (*n* = 15, NPDR); and those with proliferative retinopathy (*n* = 15, PDR); (**B**) the serum neutrophils percentage (% ± SD) in the four groups; (**C**) the Pearson correlation coefficient (r) and significance level (*p*) for the correlation of the neutrophil percentage (%) with the serum NE levels (ng/mL ± SD; r = 0.80, *p* < 0.01) in the four groups; (**D**) the serum NE levels (ng/mL ± SD) in the four groups; (**E**) the Pearson correlation coefficient and significance level for the correlation of the serum NE levels (ng/mL ± SD) with the DR stage (r = 0.86, *p* < 0.01) and (**F**) with serum AG levels (pg/mL ± SD; r = −0.73, *p* < 0.01). * *p* < 0.05 and ** *p* < 0.01 vs. CTRL; °° *p* < 0.01 vs. Diabetic; ^^ *p* < 0.01 vs. NPDR.

Serum AG was inversely associated in DR patients with serum neutrophils percentage (r = −0.74, *p* < 0.01) (Figure 3A). 

As already reported in Table 1, this was significantly increased in NPDR patients compared to the Diabetic group (*p* < 0.01) and in PDR patients vs. NPDR (*p* < 0.01 (Figure 3B). As expected, the serum neutrophil percentage positively correlated with serum NE levels (0.80, *p* < 0.01) (Figure 3C). Indeed, the serum NE was significantly increased in the Diabetic group compared to CTRL subjects (CTRL: 0.85 ± 0.3 ng/mL; Diabetic: 1.6 ± 0.4 ng/mL, *p* < 0.05 vs. CTRL) (Figure 3D). Moreover, NPDR patients showed serum NE levels that were significantly elevated compared to the Diabetic group (NPDR: 2.2 ± 0.4 ng/mL, *p* < 0.01 vs. Diabetic), with the highest value reached in PDR patients (PDR: 2.9 ± 0.6 ng/mL, *p* < 0.01 vs. NPDR) (Figure 3D). A strong positive correlation was observed between serum NE levels and DR stage (r = 0.86, *p* < 0.01) (Figure 3E), while a significant inverse association was evident between serum NE and AG (r = −0.73, *p* < 0.01) (Figure 3F). The serum levels and the correlations of neutrophils and NE in the male and female subgroups are reported in Figures S3–S5. 

## 4. Discussion

Ghrelin, often referred to as the “hunger hormone”, mediates several functions beyond its effects on appetite, food intake, body weight, and adiposity [7]. Indeed, once acylated by ghrelin-O-acyl transferase (GOAT), the 28-aa Ser3 AG binds with high affinity to the G-protein-coupled receptor GHSR1-a on central neurons and peripheral cells [39,40,41]. On these, ghrelin acts by increasing energy intake [42,43], improving cardiac functions [44,45], reducing muscle atrophy [46,47], promoting bone mass or formation [48,49], and exerting protective roles in ocular structures such as the vitreous and retina. Indeed, previously found in the eyes of embryonic chicks and rodents, ghrelin has also been localized within the nuclear compartment of the human retinal ganglion cell layer and human vitreous fluid [50,51]. In this regard, preclinical studies have evidenced antioxidant and neuroprotective actions on the retinal layers exerted by ghrelin in a rat model of glaucoma [52], as well as its modulation of retinal angiogenesis through GHSR-1a in a rat model of oxygen-induced retinopathy (OIR), where ghrelin seems to have therapeutic potential for proliferative retinopathies [51]. 

Is it worth noting that diabetic retinopathy is a complication of diabetes that damages retinal blood vessels [53]. The typical microvascular alterations that characterize this disease arise with a predictable progression, and this allows serious damage to vision to be prevented. Indeed, if neglected, diabetic retinopathy can cause severe vision loss or even blindness [54]. In the NPDR early stage, vascular occlusion and dilation occur; subsequently, the condition evolves into PDR, with the growth of new blood vessels on the retinal surface, a process known as neovascularization [54]. At the basis of these changes in the retina, there is a wide range of mediators, some pro-proliferative, others anti-proliferative. Among these is ghrelin, a peptide hormone whose endogenous levels can be compromised by pathologies such as diabetes. In fact, previous studies have found a reduced circulating ghrelin concentration in type 2 diabetes [55,56,57], both in the early stages and advanced stages with complications [58,59]. Moreover, a protective role of ghrelin was reported in DR in vitro, evidencing its anti-apoptotic and anti-inflammatory role in ARPE-19 and HMREC cells exposed to high glucose [17,18]. This was paralleled by the protective role of ghrelin on retinal layers and neovascularization in diabetic rats [17]. In line with this evidence, here, we indirectly confirm the protective role of ghrelin in DR, since diabetic patients have decreased serum levels of total ghrelin compared to non-diabetic patients, especially those with PDR respective to NPDR. However, the novelty of this research with respect to previous studies is based on the fact that, of the two known isoforms of the total ghrelin, acylated and des-acylated, the acylated form follows the reduction trend shown by the total ghrelin. Conversely, serum des-acylated ghrelin was not differentially modulated in the present clinical context, despite being the main form secreted in physiological conditions, with an AG/DAG ratio of 1:10 [60,61]. An explanation for this may be the fact that diabetes impairs the expression of enzymes that rapidly deacylate AG, such as acyl protein thioesterase 1, which normally deacylates AG [62,63]. This is in line with a recent study showing that serum AG seems to be related to elevated blood glucose levels in diabetic obese patients, while DAG seems to be involved in excess body fat mass in the same clinical setting [64]. More specifically, the present study proved that changes in serum TG in DR patients are due to AG constant decrement in NPDR and PDR patients. Particularly, serum AG levels decreased significantly in NPDR and PDR patients and showed a strong negative correlation with the DR stage. Therefore, in line with the novel clinical applications suggested for AG in Alzheimer’s and Parkinson’s disease [65], lipodystrophy [66], reproductive toxicity in cancer patients [67] and heart failure [68], serum AG could be considered a sensitive peripheral marker of distinction between NPDR and PDR.

From a mechanistic point of view, the changes in AG in DR stages mirrored the changes in, and behavior of, neutrophils, where ghrelin and its receptor are expressed [20,21]. Indeed, here, we demonstrate an increase in neutrophil percentage in DR stages, which negatively correlates with serum AG levels. During DR, neutrophils play a very important role in microangiopathy occurrence and progression, as well as in endothelial cell wall inflammatory processes [28,69], since they secrete proteolytic enzymes involved in endothelial damage [28]. This latter leads to chronic inflammation, which exacerbates microvascular complications, contributing to DR progression [28,70]. Indeed, DR patients are characterized by a high serum neutrophil count [71], with a higher neutrophil–lymphocyte ratio [71,72,73]. This correlates with DR severity in clinical settings [26,74,75]. So, to our knowledge, this is the first evidence of a possible endogenous crosstalk between AG and neutrophils for the development and progression of DR. On another note, it is well known that acting on these immune cells, ghrelin exerts anti-inflammatory effects by reducing their migration and infiltration [23,76,77,78,79]. Similarly, ghrelin has been shown to promote neutrophil apoptosis [22,78,79], which aids in the resolution of inflammation in several diseases, such as arthritis, sepsis, and respiratory pathologies [23,76,80,81,82]. 

The progression of diabetes and DR has recently been linked to elevated levels of circulating NE, a protein found within the granules of neutrophils. Studies have shown that levels of NE are higher in both type 1 and type 2 diabetic patients when compared to individuals without diabetes [29], along with PDR patients compared to the NPDR group [31]. Particularly, NE generally reflects the neutrophil count and contributes to DR pathological vascular permeability [29,32,33]. Moreover, NE is one of the constituents of NETs, along with histones, DNA, fibers, and several proteins such as myeloperoxidase (MPO), cathepsin G, cathelicidin, and proteinase 3 (PR3) [83]. NETs are a sort of physical barrier against pathogens [84,85] organized by apoptotic neutrophils during the first phase of inflammation, in a process called NETosis [86]. Although this seems to exert a protective role in an OIR mouse model, by remodeling senescence-induced pathological retinal vascularization [87], exacerbated NETosis could be incompatible with proper retinal health [87]. Indeed, besides the potential role of NETosis in exacerbating small-vessel vasculitis or microvascular occlusions due to the formation of anti-NE or anti-MPO autoantibodies found in NETs [82], both diabetes and DR progression seem to be characterized by NETs’ enhanced formation and release [29,31]. Specifically, increased neutrophils and NETs have been localized in the vitreous bodies and retinas of diabetic patients, as well as in serum from PDR patients [31]. In this regard, circulating markers of NET formation have been proposed as independent risk factors for DR [88]. In line with this evidence, here, we found a positive correlation between the increase in neutrophils in the two DR stages analyzed and the serum NE levels. These were increased in both groups, with higher levels in PDR patients compared to NPDR patients. Serum NE levels were positively correlated with the DR stage, evidencing a higher risk of NET formation, which strongly impacts the occurrence of thrombosis and hemorrhages [89,90], two of the PDR hallmarks [91]. Furthermore, here, for the first time, we show an inverse correlation between serum NE and AG in DR patients. Therefore, it could be hypothesized that the physiological role of AG in diabetes and uncomplicated retinopathy is to promote neutrophil apoptosis, consequently reducing the risk of NET formation and complications of PDR.

In conclusion, the data presented suggest that the levels of circulating ghrelin and its acylated form, along with the greater presence of retinal abnormalities and altered NE, underline the distinction between the PDR and NPDR stages. However, it is important to acknowledge the limitations of this study, including the small sample size of patients, the absence of consistent follow-up, the lack of a direct cause-and-effect study on ghrelin, and the lack of employment of a proper multivariate analysis to adjust the results for confounding factors, since a few parameters are different between groups.

## Figures and Tables

**Table 1:** Clinical characteristics of the CTRL, Diabetic, NPDR, and PDR groups.

	CTRL	Diabetic	NPDR	PDR
Female (*n*)	6	7	7	6
Male (*n*)	6	8	8	9
Mean age (years ± SD)	64.2 ± 9	65.5 ± 6	69.9 ± 7	70.1 ± 5
Age range (years)	54–74	58–74	52–82	65–77
Type I diabetes (absence of insulin) (*n*)	NA	4	6	9
Type I gender distribution (*n* of females; *n* of males)	NA	2;2	3;3	4;5
Type II diabetes (insulin resistance) (*n*)	NA	11	9	6
Type II gender distribution (*n* of females; *n* of males)	NA	5;6	4;5	2;4
Mean time from diabetes diagnosis (years ± SD)	NA	6.0 ± 0.8	6.8 ± 1	7.8 ± 1.1
Mean time from DR diagnosis (years ± SD)	NA	NA	2.6 ± 0.2	3.1 ± 0.4
Glycaemia (mg/dL) normal range (70–100 mg/dL)	82 ± 15	140.2 ± 25 **	142.4 ± 20 **	200 ± 25 °^
Neutrophils (% ± SD) normal range (40–70%)	43.9 ± 2	45.4 ± 4	50.8 ± 5 °°	61.2 ± 3 °°^^

NA: not applicable; CTRL: non-diabetic subjects with absence of ocular pathologies (*n* = 12); Diabetic: diabetic patients without DR signs (*n* = 15); NPDR: diabetic patients with diagnosis of non-proliferative DR (*n* = 15); PDR: diabetic patients with diagnosis of proliferative DR (*n* = 15). ** *p* < 0.01 vs. CTRL; ° *p* < 0.05 and °° *p* < 0.01 vs. Diabetic; ^ *p* < 0.05 and ^^ *p* < 0.01 vs. NPDR. Control, Diabetic, NPDR, PDR.

## Data Availability

All the data presented in this study are included within the article and its Supplementary Material.

## Supplementary Materials

The following supporting information can be downloaded at: https://www.mdpi.com/article/10.3390/medicina60010118/s1, Figure S1: Serum levels of total, acylated, and des-acylated ghrelin in male and female subgroups; Figure S2: Serum acylated/des-acylated ratio and its correlation with DR stage in male and female subgroups; Figure S3: Serum neutrophil percentage and its correlation with acylated ghrelin in male and female subgroups; Figure S4: Serum neutrophil elastase and its correlation with neutrophil percentage in male and female subgroups; Figure S5: Correlation of serum neutrophil elastase with DR stage or acylated ghrelin in male and female subgroups.
