# Supplementary material for: Changes in Circulating Acylated Ghrelin and Neutrophil Elastase in Diabetic Retinopathy
Source: Medicina (Kaunas). 2024 Jan 8;60(1):118. doi: 10.3390/medicina60010118 (PMC10820226; doi:10.3390/medicina60010118)
Supplement: Supplementary file 1 [file medicina-60-00118-s001.zip › medicina-2743834-supplementary.pdf]

medicina

MDPI

Article

Changes of circulating acylated ghrelin and neutrophils elastase in diabetic retinopathy

Maria Consiglia Trotta [1,†], Carlo Gesualdo [2,†], Marina Russo [3,4], Caterina Claudia Lepre [1,5], Francesco Petrillo [1,5], Maria Giovanna Vastarella [5], Maddalena Nicoletti [2], Francesca Simonelli [2], Anca Hermenean [6], Michele D'Amico [1,‡] and Settimio Rossi [2,*,‡]

1 Department of Experimental Medicine, University of Campania "Luigi Vanvitelli", 80138 Naples, Italy; mariaconsiglia.trotta2@unicampania.it (M.C.T.); caterinaclaudia.lepre@unicampania.it (C.C.L.); francesco.petrillo@unicampania.it (F.P.); michele.damico@unicampania.it (M.D.)
2 Multidisciplinary Department of Medical, Surgical and Dental Sciences, University of Campania "Luigi Vanvitelli", 80138 Naples, Italy; carlo.gesualdo@unicampania.it (C.G.); mnicoletti2023@libero.it (M.N.); francesca.simonelli@unicampania.it (F.S.)
3 PhD Course in National Interest in Public Administration and Innovation for Disability and Social Inclusion, Department of Mental, Physical Health and Preventive Medicine, University of Campania "Luigi Vanvitelli", 80138 Naples, Italy; marina.russo@unicampania.it
4 School of Pharmacology and Clinical Toxicology, University of Campania "Luigi Vanvitelli", 80138 Naples, Italy
5 PhD Course in Translational Medicine, University of Campania "Luigi Vanvitelli", 80138 Naples, Italy; mariagiovanna.vastarella@unicampania.it
6 "Aurel Ardelean" Institute of Life Sciences, Vasile Goldis Western University of Arad, 310144 Arad, Romania; hermenean.anca@uvvg.ro
* Correspondence: settimio.rossi@unicampania.it
† These authors contributed equally to this work as first authors.
‡ These authors contributed equally to this work as last authors.

Supplementary Material

Supplementary Figures

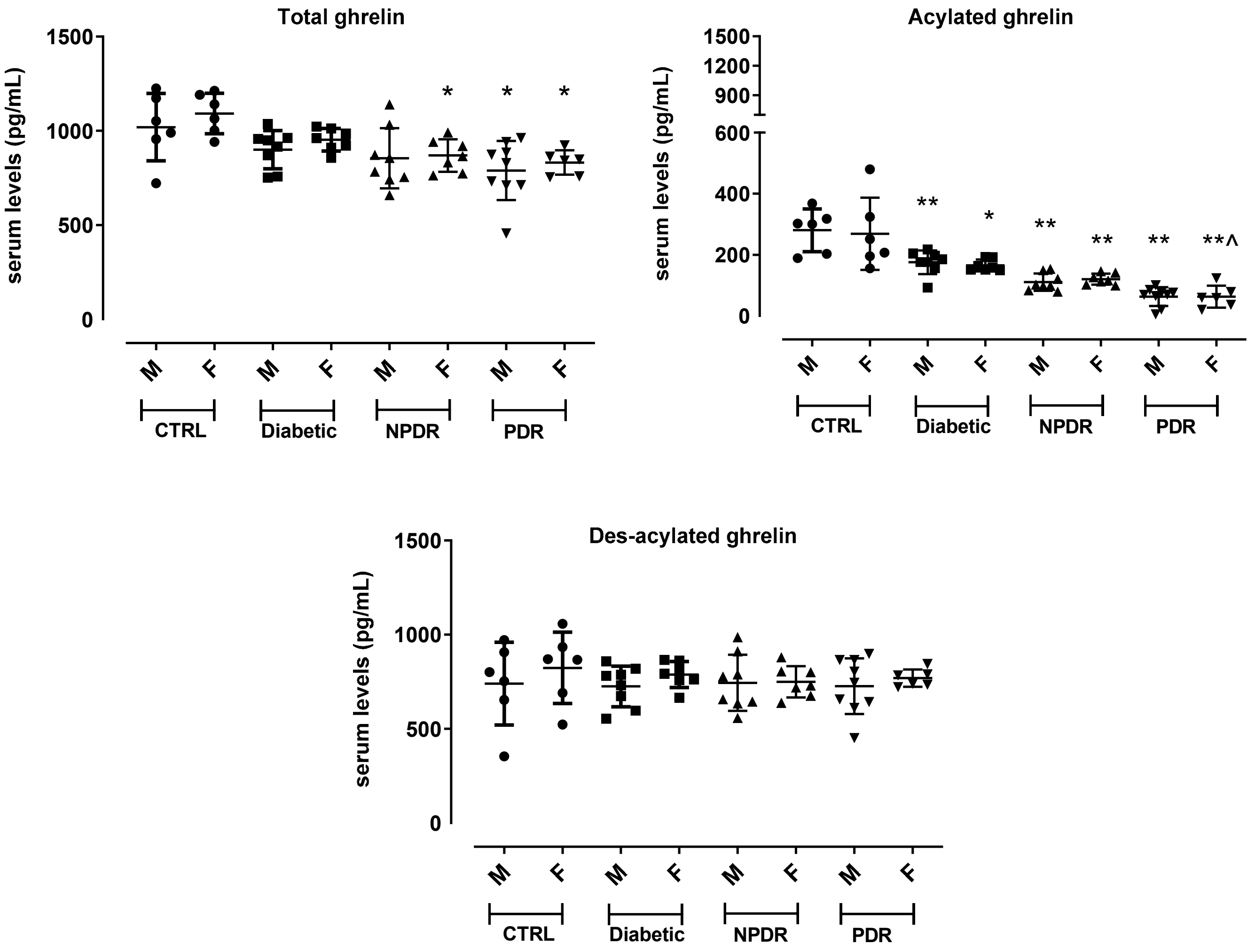

Figure S1. Serum levels of total, acylated and des-acylated ghrelin in male and female subgroups. Non-diabetic subjects with absence of ocular pathologies (6 M and 6 F - CTRL); diabetic patients with no signs of diabetic retinopathy (8 M and 7 F - Diabetic); diabetic patients with non-proliferative diabetic retinopathy (8 M and 7 F - NPDR) or proliferative retinopathy (9 M and 6 F - PDR); M = males, F = females; *P < 0.05 and ** P < 0.01 vs CTRL, same sex; ^ P < 0.05 vs NPDR, same sex.

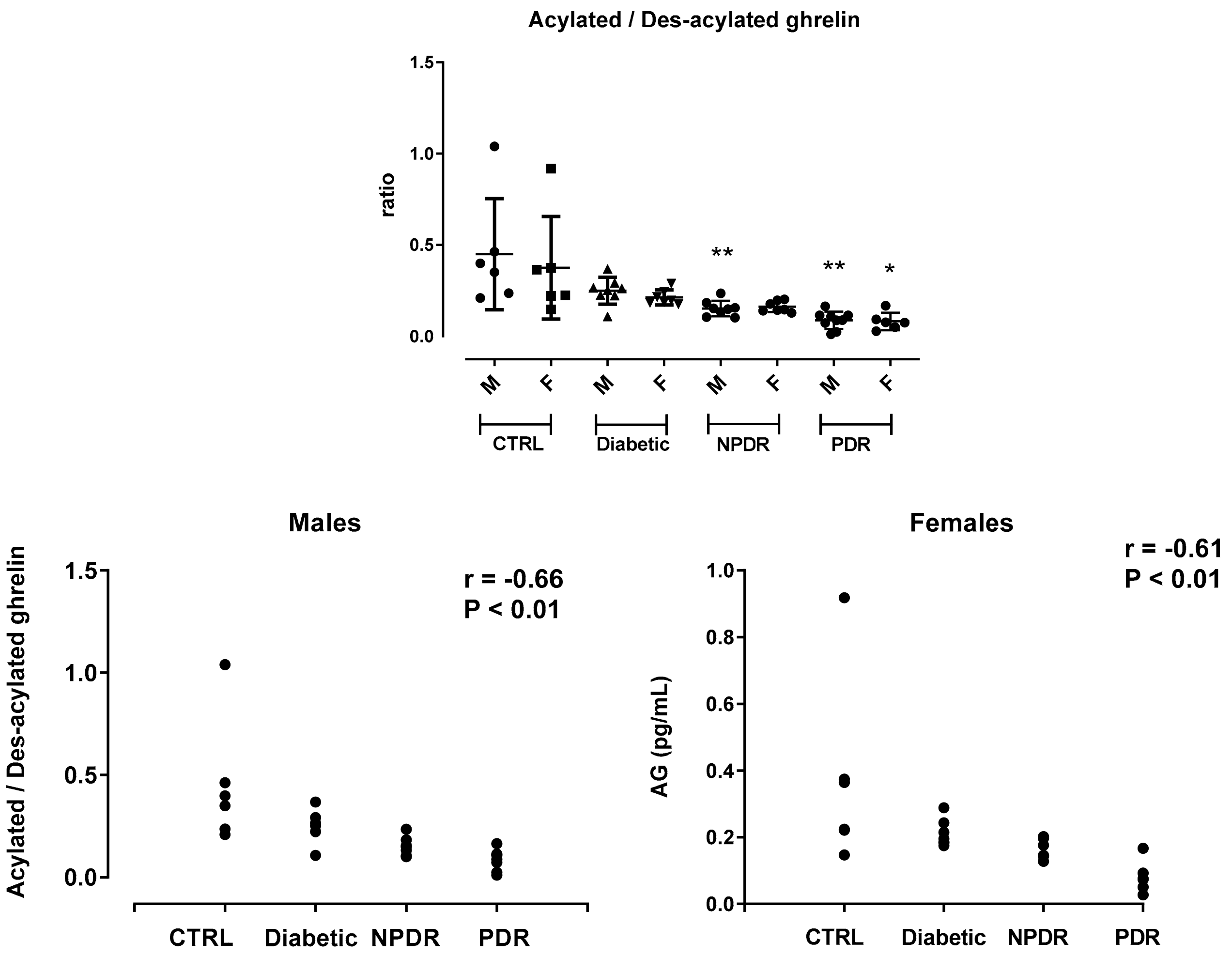

Figure S2. Serum Acylated/Des-acylated ratio and its correlation with DR stage in male and female subgroups. Non-diabetic subjects with absence of ocular pathologies (6 M and 6 F - CTRL); diabetic patients with no signs of diabetic retinopathy (8 M and 7 F - Diabetic); diabetic patients with non-proliferative diabetic retinopathy (8 M and 7 F - NPDR) or proliferative retinopathy (9 M and 6 F - PDR). M = males, F = females; * P < 0.05 and ** P < 0.01 vs CTRL, same sex.

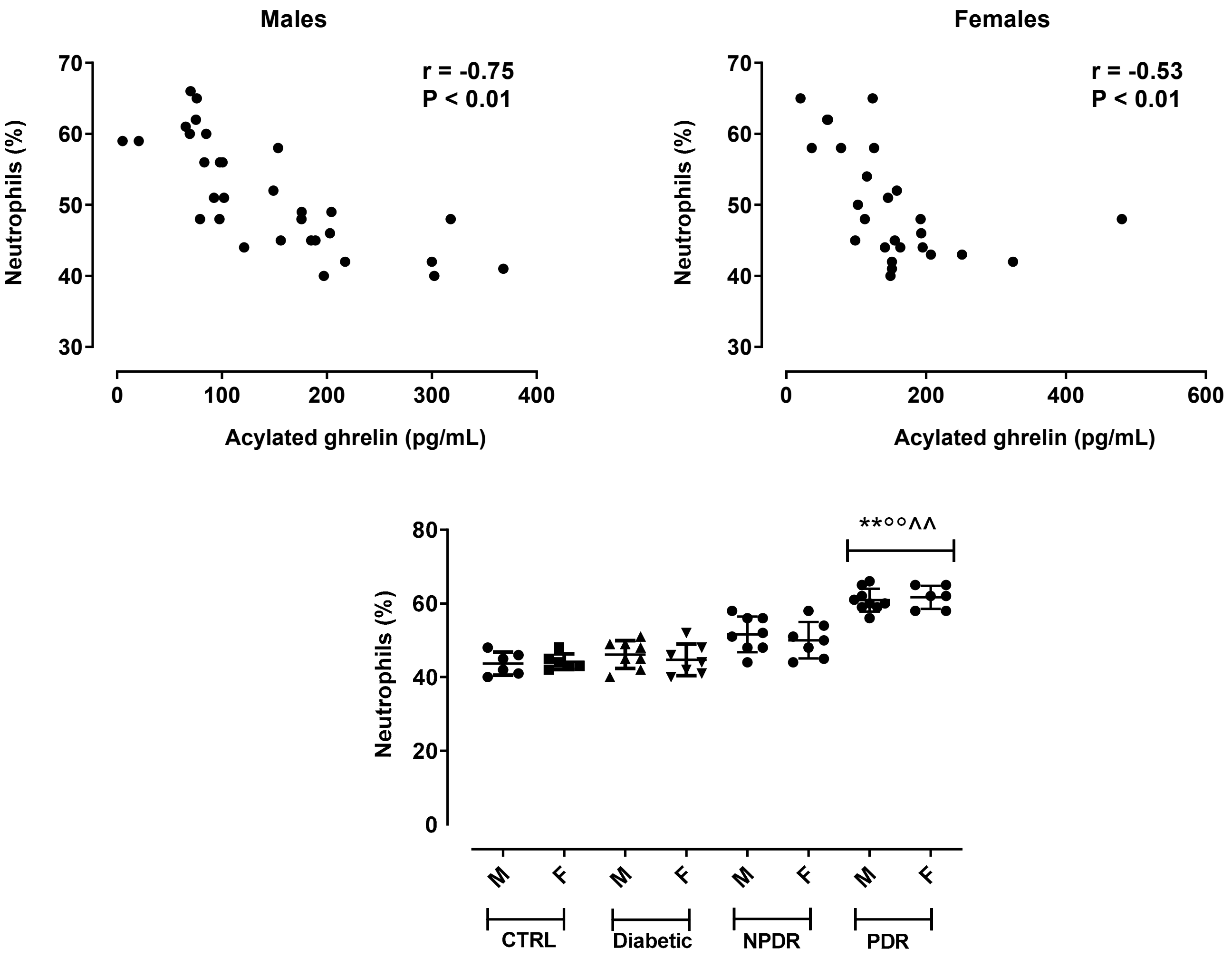

Figure S3. Serum neutrophils percentage and its correlation with Acylated ghrelin in male and female subgroups. Non-diabetic subjects with absence of ocular pathologies (6 M and 6 F - CTRL); diabetic patients with no signs of diabetic retinopathy (8 M and 7 F - Diabetic); diabetic patients with non-proliferative diabetic retinopathy (8 M and 7 F - NPDR) or proliferative retinopathy (9 M and 6 F - PDR). M = males, F = females; ** $P < 0.01$ vs CTRL, same sex; °° $P < 0.01$ vs Diabetic, same sex; ^^ $P < 0.01$ vs NPDR, same sex.

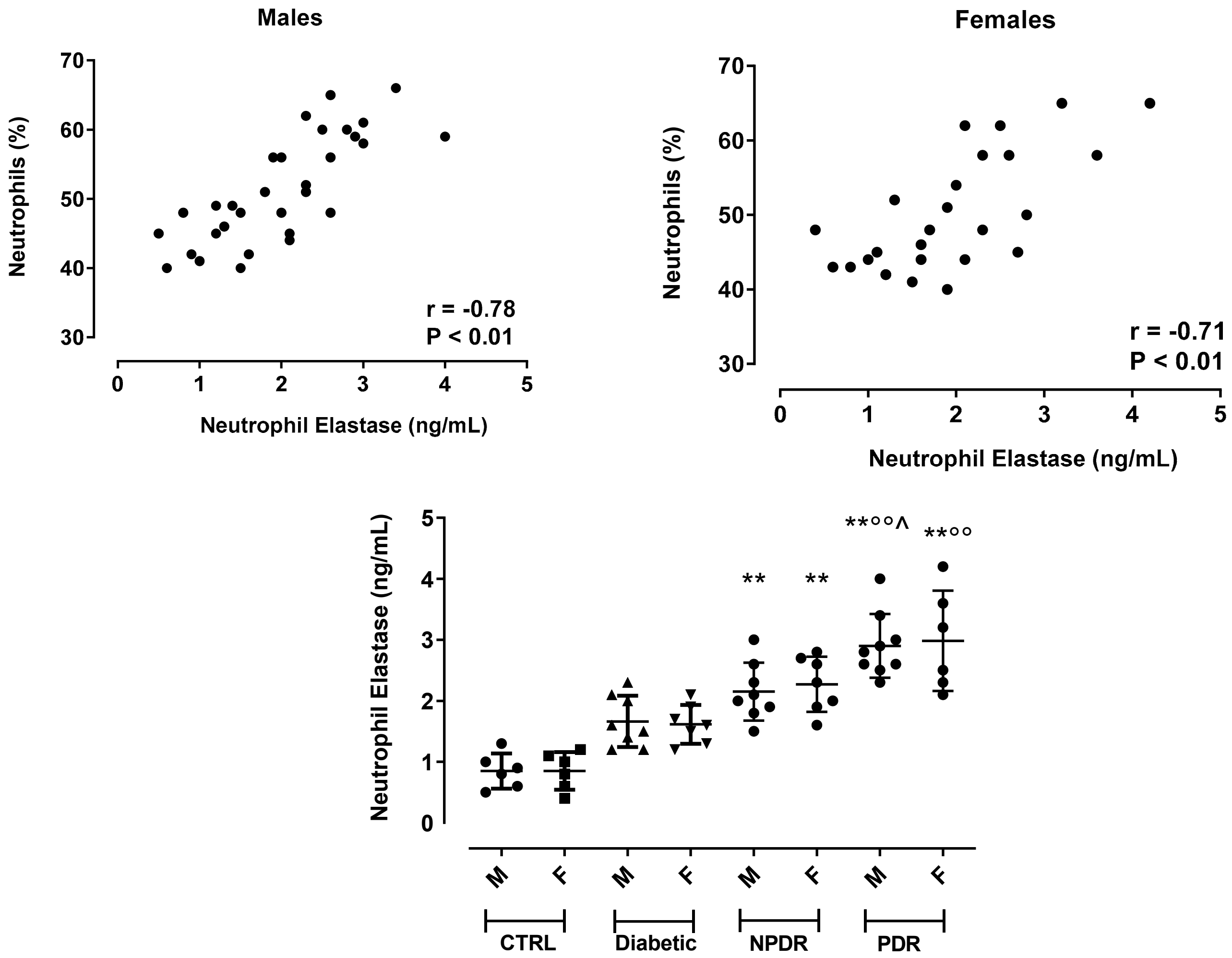

Figure S4. Serum Neutrophil Elastase and its correlation with Neutrophils percentage in male and female subgroups. Non-diabetic subjects with absence of ocular pathologies (6 M and 6 F - CTRL); diabetic patients with no signs of diabetic retinopathy (8 M and 7 F - Diabetic); diabetic patients with non-proliferative diabetic retinopathy (8 M and 7 F - NPDR) or proliferative retinopathy (9 M and 6 F - PDR). M = males, F = females; ** P < 0.01 vs CTRL, same sex; °° P < 0.01 vs Diabetic, same sex; ^ P < 0.05 vs NPDR, same sex.

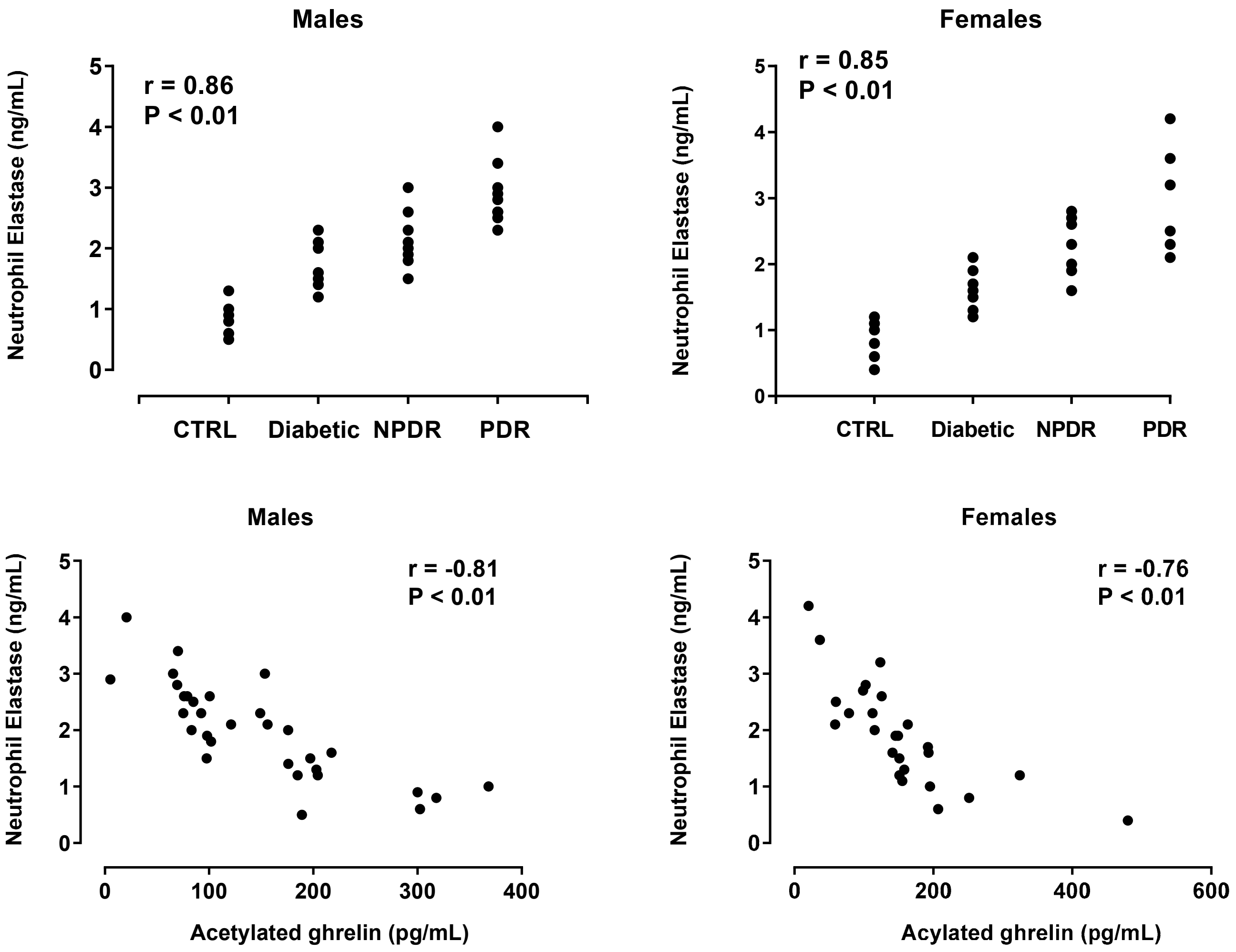

Figure S5. Correlation of serum Neutrophil Elastase and DR stage or Acylated ghrelin in male and female subgroups. Non-diabetic subjects with absence of ocular pathologies (6 M and 6 F - CTRL); diabetic patients with no signs of diabetic retinopathy (8 M and 7 F - Diabetic); diabetic patients with non-proliferative diabetic retinopathy (8 M and 7 F - NPDR) or proliferative retinopathy (9 M and 6 F - PDR).
